# Accelerating the Western Pacific tobacco endgame

**DOI:** 10.1016/j.lanwpc.2026.102018

**Published:** 2026-10-30

**Authors:** The Lancet Regional Health – Western Pacific

Tobacco control was one of the key themes at this year's World Cancer Congress in September 2026, in Hong Kong, China. Discussions were centred on innovative tobacco cessation and control measures, new nicotine products, and industry interference. These issues are particularly relevant to the Western Pacific region, which has the largest population of tobacco users, but the slowest rate of reduction in tobacco use among all WHO regions.

Despite the declining smoking prevalence, most countries in the Western Pacific region are off-track to achieve the 30% reduction target in tobacco use from the 2015 baseline by 2030. According to the latest situation analysis released during the 77th session of the WHO Regional Committee for the Western Pacific this October, MPOWER scores (a policy package contains Monitor, Protect, Offer, Warn, Enforce, and Raise) have been stagnant across the region over time, reflecting the stalled implementation of the key demand-reduction measures. Tobacco industry interference scores show a worsening trend, indicating greater tobacco industry interference and weak government responses. Youth e-cigarette use exceeds adult prevalence in countries where data for both measures are available, picturing a youth-targeting e-cigarette epidemic in the region. Therefore, the 2026 acceleration plan highlights three priorities for actions: increase tobacco taxes, close implementation gaps, and regulate new and emerging tobacco and nicotine products to prevent youth uptake.

Countries in the region face different challenges in tobacco taxation. In countries where tobacco industries contribute substantially to national revenue or economic activity, there are tensions between public health objectives and economic considerations. Raising tobacco taxation is not easy but is the right thing to do; population health should not be traded for short-term economic gains. On the one hand, governments need to find a way to pair tax reform with measures that counter industry influence and support a transition away from dependence on tobacco revenue. On the other, countries with the highest cigarette taxes in the world, such as Australia, New Zealand, and Singapore, face the challenge of how the impact of such taxes can be sustained. The Coalition Government of Australia recently unveiled a plan to wind back tobacco taxes, with the aim to kill the illicit tobacco market. Reducing cigarette prices could increase smoking and reverse decades of tobacco-control gains in the country. Sustaining taxation policy therefore requires both firm political commitment and public support, as well as a willingness to confront misinformation that can weaken proven tobacco-control measures.

Accelerating progress also requires countries to close implementation gaps within proven high-impact measures, including cessation support, smoke-free policies, warning labels, and advertising bans. Innovative tools such as personalised text messages and chat-based interventions to provide cessation support are promising. However, the success of these initiatives largely depends on individuals' willingness and commitment to ending tobacco use. One potential approach to encouraging tobacco cessation is to amplify awareness that an individual's tobacco-use behaviour not only harms themselves but also imposes preventable health risks, caregiving burdens, and increased health-care costs on others, particularly their loved ones. Younger children, older parents, and family members with chronic respiratory or cardiovascular diseases are all vulnerable to secondhand or thirdhand smoke. However, their health interests remain insufficiently represented in tobacco-control policies and cessation discussions. More proactive measures and clearer communications are necessary to increase the visibility of individuals affected by tobacco who are often overlooked. For example, add warning signs on products to emphasise the externalised harm to families and communities, increase public knowledge on secondhand and thirdhand smoke, and encourage smoking bans in outdoor public spaces, homes, and vehicles.

Emerging tobacco and nicotine products, including e-cigarettes (ie, vapes) and heated tobacco products, threaten the tobacco endgame in the region. Through narratives of harm reduction, the tobacco industry is creating the illusion that a smoke-free world is a healthier one. By promoting new smokeless tobacco and nicotine products, it might influence a new generation of youth into becoming smoke-free smokers. However, emerging evidence shows that smokeless products can expose users to higher risks of cancers, cardiovascular and respiratory diseases, adverse pregnancy outcomes, and can affect brain development in children and adolescents. Studies from Japan, New Zealand, and Australia have consistently shown that although new nicotine products are displacing conventional cigarette smoking in some populations (especially in youth populations), they are not eliminating nicotine use and could create a new generation of nicotine addiction. The number of cigarette and novel nicotine product dual users, new tobacco users among never smokers, and individuals who transitioned from cigarettes to new products remain high. There have been few cessation initiatives or strategies for new nicotine products, while some are even used as substitutes in cigarette cessation programmes. To protect tobacco-control gains, we need to address new nicotine and tobacco products within established tobacco-control frameworks and treat them identically to traditional cigarettes.

Accelerating the tobacco endgame in the Western Pacific will require the region to move beyond a narrow focus on smoking cessation. The goal is not merely to have a smoke-free world, but a tobacco-free one.

